# Synthesis and application of magnetite dextran-spermine nanoparticles in breast cancer hyperthermia

**DOI:** 10.1007/s40204-017-0068-8

**Published:** 2017-06-17

**Authors:** Reza Avazzadeh, Ebrahim Vasheghani-Farahani, Masoud Soleimani, Saeid Amanpour, Mohsen Sadeghi

**Affiliations:** 1 Biomedical Engineering Group, Faculty of Chemical Engineering, Tarbiat Modares University, Tehran, Iran; 2 Hematology Group, Faculty of Medical Sciences, Tarbiat Modares University, Tehran, Iran; 3 Cancer Biology Research Center, Tehran University of Medical Sciences, Tehran, Iran

**Keywords:** Cancer hyperthermia, Anti-HER2, Magnetic nanoparticles, Dextran-spermine

## Abstract

Cancer treatment has been very challenging in recent decades. One of the most promising cancer treatment methods is hyperthermia, which increases the tumor temperature (41–45 °C). Magnetic nanoparticles have been widely used for selective targeting of cancer cells. In the present study, magnetic dextran-spermine nanoparticles, conjugated with Anti-HER2 antibody to target breast cancer cells were developed. The magnetic dextran-spermine nanoparticles (DMNPs) were prepared by ionic gelation, followed by conjugation of antibody to them using EDC-NHS method. Then the Prussian blue method was used to estimate the targeting ability and cellular uptake. Cytotoxicity assay by MTT showed that antibody-conjugated MNPs (ADMNPs) have no toxic effect on SKBR3 and human fibroblast cells. Finally, the hyperthermia was applied to show that synthesized ADMNPs, could increase the cancer cells temperature up to 45 °C and kill most of them without affecting normal cells. These observations proved that Anti-HER2 conjugated magnetic dextran-spermine nanoparticles can target and destroy cancer cells and are potentially suitable for cancer treatment.

## Introduction

Breast cancer is a malignant tumor that originates from healthy mammary gland cells which is most frequent among women aged between 50 and 70. It is the most common type of cancer among women which affects one in eight women on average (Alphandery 2014; Matsen and Neumayer 2013; Tinoco et al. 2013). Surgery, chemotherapy and radiotherapy are the most common strategies to treat breast cancer. These conventional cancer therapies, having limitations such as toxic side effects and drug resistance, have often failed to completely eliminate the tumor (Chalkidou et al. 2011; Chen et al. 2014; Debnath et al. 2016; Sahu et al. 2013).

Hyperthermia is the application of heat to destroy the tumor cells or sensitize them to drugs and radiation by increasing blood flow and inducing immune responses. The tumor cells, by having poorly developed vessels and nervous system, so insufficient oxygen supply and inability to dissipate heat, are sensitive to temperatures in the range of 41–45 °C, which damages tumor cells irreversibly, while normal cells can tolerate even higher temperatures (Chen et al. 2009; Kawashita et al. 2005; Lin et al. 2012; Meenach et al. 2010; Purushotham and Ramanujan 2010; Rao et al. 2013; Sen et al. 2011). An adequate amount of heat should be delivered to the tumor so that it reaches the desired temperatures, or it may induce resistance. The magnetic fluid hyperthermia is a non-invasive method which can deliver the desired heat to deep-seated tumors without damaging healthy tissues. It predominantly uses superparamagnetic iron oxide nanoparticles (SPIONs) which are biocompatible, non-toxic and easy to synthesize. They have raised great attention in biomedical applications such as magnetic resonance imaging, drug delivery and magnetic hyperthermia where SPIONs produce heat in an alternating magnetic field (AFM) thorough either Néel or Brownian relaxation (Lin et al. 2012; Ma et al. 2004; Sadhukha et al. 2013; Stocke et al. 2016; Yallapu et al. 2011).

The magnetic particles can be introduced into the body via the systemic circulation and guided to the target site under the influence of magnetic field or via direct injection into the tumor. However, in case of intravenous injection, the particle size should be controlled as well as surface modification in a way that inhibits protein adsorption and phagocytosis. SPIONs, having a hydrophobic nature, tend to aggregate, so have a short half-life in blood circulation, leading to poor bioavailability. To resolve this challenge, polymeric and inorganic nanoparticles, liposomes, micelles and phospholipid complexes have been used to encapsulate them for delivery. Among them, polymeric nanoparticles are emerging as one of the best options due to their higher stability (Cole et al. 2011a; Lin et al. 2012; Liu et al. 2011; Rao et al. 2013). The particles can be delivered to the tumor either passively thorough vascularization and the enhanced permeation and retention effect (EPR) or actively thorough receptor-mediated endocytosis. However, active targeting results in higher local concentrations of nanoparticles and lower systemic concentrations, which is essential for more effective treatment (Cole et al. 2011; Kruse et al. 2014; Lin et al. 2012; Ling et al. 2017; Ruoslahti et al. 2010; Wuang et al. 2008).

In this study an atni-HER2 conjugated dextran-spermine magnetic nanoparticle was developed for breast cancer magnetic hyperthermia. Atni-HER2 is a humanized IgG monoclonal antibody directed against the extracellular domain of the human epidermal growth factor receptor 2 (HER-2), which is overexpressed in some types of breast cancer cells. The antibody can be efficient in cell uptake of the carrier system and nanoparticles thorough the internalization ability of Anti-HER-2 (Moore and Cobleigh 2007; Wuang et al. 2008). First, dextran-spermine was synthesized and the amine content was evaluated as described in the literature (Azzam et al. 2002). The dextran-spermine magnetic nanoparticles (DMNPs) were prepared using ionic gelation method. The size, zeta potential and morphology of the nanoparticles were analyzed. Finally, the antibody was conjugated to the nanoparticles. The anti-HER2 conjugated dextran-spermine magnetic nanoparticles (ADMNPs) were characterized and compared to DMNPs and iron oxide nanoparticles (MNPs) in terms of biocompatibility, cell uptake and in vitro hyperthermia for cancerous SKBR3 and normal fibroblast cells.

## Materials and methods

### Dextran-spermine synthesis

**Fig. 1:**
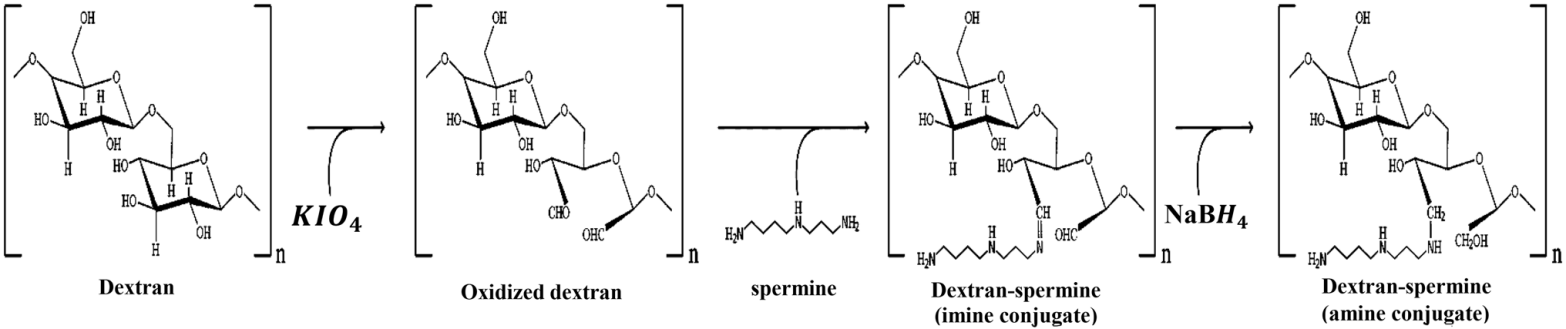
Dextran-spermine formation

Dextran-spermine conjugate was prepared according to Azzam method (Azzam et al. 2002). Briefly, 10 g dextran was dissolved in 200 mL doubly deionized water (DDW); and potassium periodate at molar ratio of 1:1 was added to the dextran solution. The resulting solution was stirred for 8 h in a dark room to form a clear-yellow solution. This solution was dialyzed for 2 days against DDW in a cellulose tube with 12,000 Dalton MW cutoff at 4 °C while the dialysis water was refreshed every 6 h. Then the purified solution was freeze-dried to obtain a white powder (oxidized dextran). A solution of 0.57 g of oxidized dextran in 57 mL of DDW was slowly (during 5 h) added to a solution containing 1.25 equimolar amount of the spermine dissolved in 100 mL of 0.1 M borate buffer. The resulting solution was stirred for 24 h at room temperature, then dialyzed against DDW in a cellulose tube with 3500 Dalton MW cutoff at 4 °C. The amine-based conjugates were obtained after reducing the imines’ conjugates by adding 1 g NaBH4 to the mixture at room temperature and stirring for 48 h. The reduction was repeated by adding another 1 g NaBH4 and stirring for 24 h. The resulting solution was dialyzed again using a 3500-MW cutoff tubing (Membrane Filtration Products Inc., San Antonio, TX, USA), then freeze-dried to obtain a yellow amine-based conjugate powder. The synthesized polycation was characterized for its primary amine content and structure using TNBS method (Mohammad-Taheri et al. 2012) and H-NMR analysis. Figure1 shows the schematical dextran-spermine synthesis route.

### Characterization of the synthesized polymer

H-NMR analysis and TNBS method were used to determine the formation of the primary amine groups. For TNBS method, dextran-spermine was dissolved in 0.1 M sodium bicarbonate solution (pH 8.5) to obtain solutions ranging 20–200 µg/mL. A standard calibration curve for l-lysine was plotted. 1% TNBS was added to 0.1 M sodium bicarbonate solution and 500 µg of the resulting solutions was added to 1 mL of each sample. The samples were incubated for 2 h at 37 °C. Finally, 500 µg sodium dodecyl sulfate (10% w/v) and 250 µL HCl (1 N) were added to each sample to stop the reaction. The absorbance of the samples was recorded at 345 nm using a spectrophotometer (UV–Vis-CARY50). The primary amine content was calculated according to the calibration curve. H-NMR spectra were recorded on a BRUKER DRX500 AVANCE (500 MHz) instrument using DDW as solvent. Values were recorded as ppm relative to internal standard (TMS).

### Preparation of SPION-loaded dextran-spermine nanoparticles

**Fig. 2:**
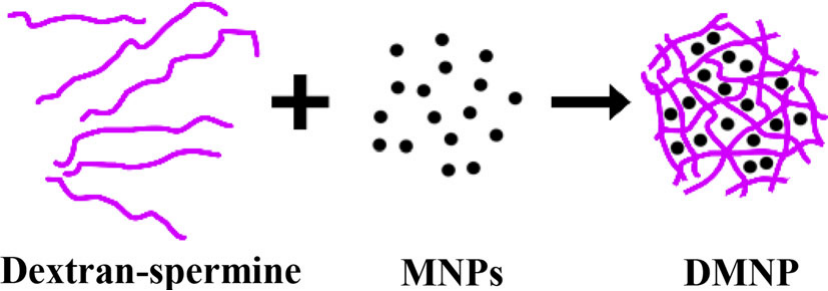
DMNPs formation

The MNP-loaded dextran-spermine nanoparticles (DMNP) were prepared by ionic gelation technique as shown in Fig. 2. In brief, 5 mg SPION (MNP) was added to 2 mL of a 2.92 mg/mL dextran-spermine solution and sonicated for 5 min. The cationic dextran nanoparticles were formed upon addition of 800 µL of an aqueous tripolyphosphate (TPP) solution to the above mentioned suspension under mechanical stirring. Finally, the suspension was centrifuged first at 5000*g* for 3 min to separate larger particles and then at 30,000*g* for 30 min to remove free MNPs and isolate the nanoparticles with desired size (80–100 nm).

### Characterization of the MNP-loaded nanoparticles

DLS analysis was carried out to determine the size distribution and zeta potential of the DMNP using a particle size analyzer (PSA) (Malvern, 3000 HAS, England). The DMNPs were suspended in purified water and sonicated to produce a homogenous suspension for measurement. The zeta potential of the nanoparticles was also measured to confirm their surface charge. TEM imaging (Zeis-EM10C-80 kV) was also used to evaluate the size, morphology and encapsulation of the nanoparticles. A drop of well-dispersed nanoparticle suspension was placed on a copper grid and then dried at ambient condition before its attachment to the sample holder of the microscope.

### Conjugation of the antibody to the MNP-loaded nanoparticles

**Fig. 3:**
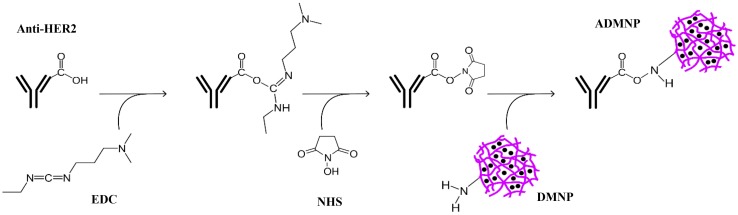
Conjugation of anti-HER2 to DMNPs using EDC-NHS

The antibody was conjugated to the nanoparticles using EDC-NHS (Fischer 2010) as shown schematically in Fig. 3. In this method, the carboxyl group at the surface of the antibody was activated using EDC-NHS to form an active ester group which would bond to the amine group at the surface of the nanoparticles (Hermanson 2013). In brief, the antibody was dissolved in a reaction buffer (pH 6) containing MES 0.05 M and NaCl 0.5 M. Then EDC (2 mM) and NHS (5 mM) were added to the mixture. After stirring for 15 min at room temperature, 1 µL mercaptoethanol was added to stop the reaction. The MNP-loaded nanoparticles, suspended in a MES buffer at pH 7.5, were added after 10 min of further stirring. The final suspension was stirred for 2 h at room temperature. Finally the antibody-conjugated nanoparticles were centrifuged, separated and redispersed in DDW.

### Characterization of antibody-conjugated nanoparticles

Size distribution and zeta potential of antibody-conjugated MNP-loaded dextran-spermine nanoparticles (ADMNP) were also measured using a particle size analyzer. Comparing these results to those of the DMNP can confirm the antibody conjugation. Antibody conjugation to the nanoparticles was also evaluated using Bradford assay. The Bradford solution was prepared by adding 100 mg of Coomassie brilliant blue G250 to 50 mL ethanol, then mixing by 100 mL phosphoric acid (85% w/w) and finally adding them to DDW to reach the volume of 1 L. Then 5 mL of the solution was added to different concentrations of the antibody ranging from 0 to 1 mg/mL and their absorbances at 600 nm were measured. These values were plotted to form a standard curve. The amount of the antibody in the supernatant of the centrifuged antibody-conjugated nanoparticles was determined by comparing to the standard curve.

### Cell culture

Immortalized primary human fibroblast and breast cancer SKBr3 cells obtained from cell bank (Stem cell technology research center, Tehran, Iran) were grown in T75 cell culture flasks in 3 mL of complete medium. The medium consisted of Dulbecco’s Modified Eagles Medium (DMEM), with 10% of fetal bovine serum (FBS) and Penicillin/Streptomycin. The cells were incubated in an incubator (RS Biotech Galaxy) at 37 °C with a 5% CO2 atmosphere until they reach the suitable confluence.

### Cytotoxicity assay

In vitro cytotoxicities of MNPs, DMNPs and ADMNPs against breast cancer SKBR3 and normal fibroblast cells were evaluated based on the MTT (3-[4,5-dimethylthiazol-2-yl]-2,5-diphenyltetrazolium bromide) assays. SKBR3 and human fibroblast cells with cell density of 5000 per well were dispensed in 96-well plates in triplicate, and 200 µL of fresh medium was added to each well and incubated for 48 h. Then, the cells were treated with different concentrations of the nanoparticles (50, 80 and 150 µg/mL medium). A control group containing no particle was also considered for each cell type. The samples were incubated for 24 and 48 h. After the incubation, the wells were washed with 200 µL PBS and overlaid with 50 µL fresh media containing 0.5 mg/mL of MTT reagent and incubated for 4 h. Subsequently, the media were aspirated and formazan crystals were solubilized in 200 µL dimethyl sulfoxide (DMSO). The averaged absorbance at 570 nm for each treatment group was normalized to the zero-time viability values.

### Uptake assay

Cellular uptake was evaluated using iron staining method. For this purpose, the cultured SKBR3 and fibroblast cells were seeded in 96-well plates with cell density of 5000 per well and 200 µL of fresh media was added. The cells were left to incubate for 24 h, prior to addition of nanoparticles. Three sample groups (MNP, DMNP and ADMNP) and a control were considered for each cell type. The samples were overlaid by 200 µL of fresh media containing 80 µg/mL nanoparticles and after 6 h of incubation, the culture medium was removed and the cells were fixed upon addition of 500 µL 10% neutral buffered formalin. After 20 min, the formalin was replaced by 500 µL Prussian blue solution, containing 5% w/v potassium ferrocyanide and 5% v/v HCl and left till blue stains appeared. Prussian blue is a prototype of mixed-valence transition-metal hexacyanoferrates with the general formula of Fe_4_[Fe(CN)_6_]_3_ (Cheng et al. 2014). The cells then were washed twice by PBS and examined by microscope.

### Hyperthermia study

For in vitro hyperthermia study, SKBR3 and fibroblast cells were seeded in the 96-well plates with the concentration of 5000 cells per well and 200 µL of fresh media was added. After 48 h of incubation, the media was replaced by 200 µL of fresh media containing optimum concentration of 80 µg/mL (Attar et al. 2016) of three magnetic nanoparticle groups (MNP, DMNP and ADMNP). A control group with no magnetic substance was also considered for each cell type. After 8 h of incubation, the wells were washed with 200 µL PBS and overlaid with 200 µL fresh media. The cells were left to incubate overnight prior to initiating the test. The samples were placed in the copper coil of a radio frequency generator at 80 kHz frequency with 150 kA/m field. The temperature was monitored over the test.

### Statistical analysis

All experiments were performed at least in triplicate. Microsoft office excel was used for *t* tests (paired *t* test with unequal variances) to determine any significance in the observed data. The *P* value <0.05 was considered statistically significant.

## Results and discussion

### Characterization of the synthesized polymer

**Fig. 4:**
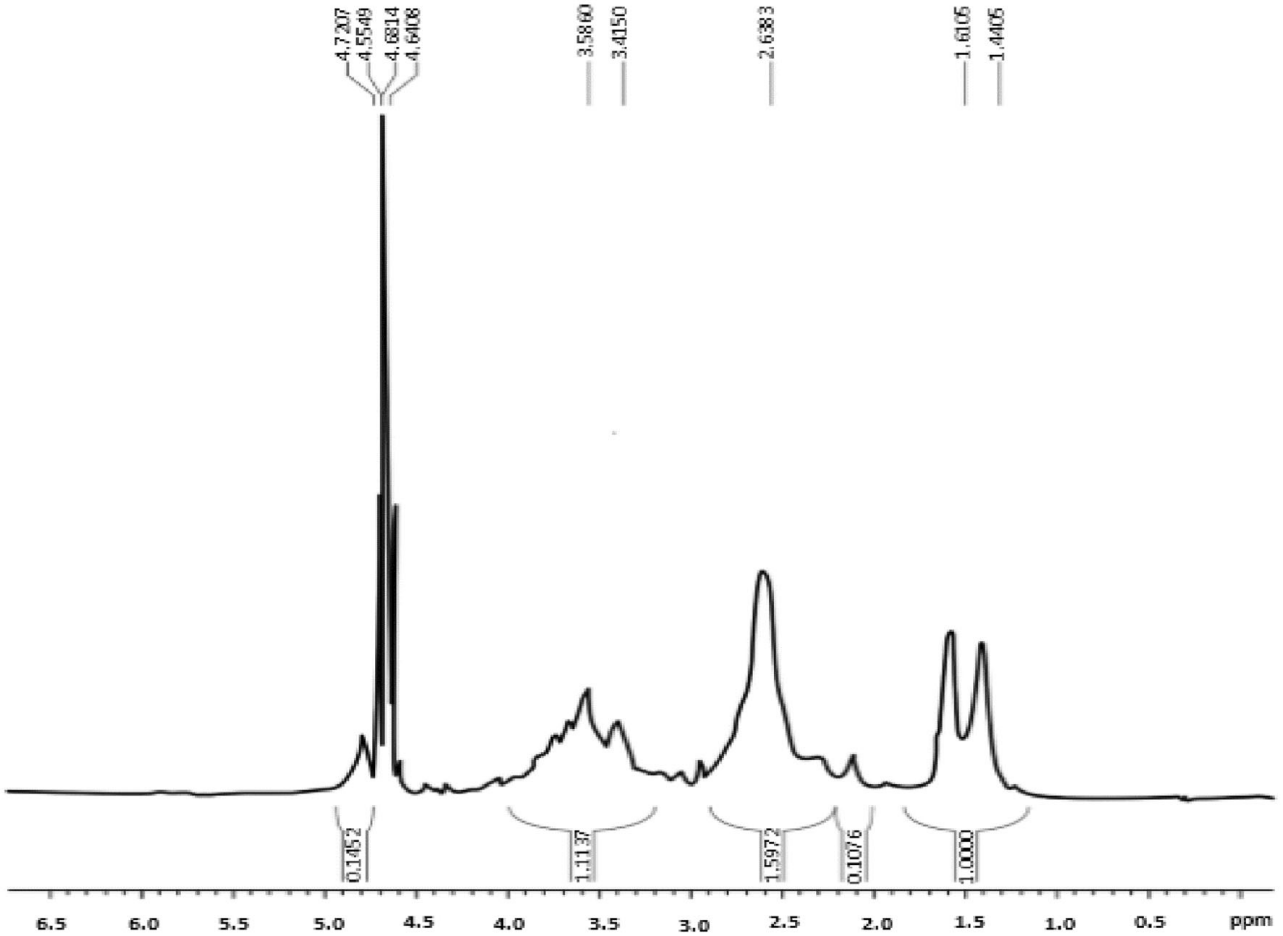
H-NMR spectra of synthesized dextran-spermine

The primary amine content of the synthesized dextran-spermine conjugate was determined according to an l-lysine standard curve. For the dextran-spermine solution an absorbance of 0.577 was read; according to the curve, this absorbance is equivalent to the concentration of 19.75 µg/mL of l-lysine. So considering that l-lysine has 2 amine groups, the primary amine group content for synthesized dextran-spermine was 1.43 mM/g that is within the range of previous studies (Mohammad-Taheri et al. 2012; Tarvirdipour et al. 2016). The structure of the synthesized polymer was evaluated using H-NMR analysis (Fig. 4). The H-NMR spectra were as follows:

1.4405 (m, 4H, dextran-CH_2_ NH (CH_2_)_3_ NH CH_2_CH_2_ CH_2_CH_2_ NH (CH_2_)_3_ NH_2_), 1.6106 (m, 4H, dextran-CH_2_ NH CH_2_CH_2_CH_2_ NH (CH_2_)_4_NH CH_2_CH_2_CH_2_ NH_2_), 2.6303 (m, 14H, dextran-CH_2_ NH CH_2_CH_2_CH_2_ NH CH_2_CH_2_CH_2_CH_2_ NH CH_2_CH_2_CH_2_ NH_2_), 3.4150–3.5860 (m, glycoside hydrogens) and 4.6822 (m, 1H, anomeric hydrogen).

### Characterization of the MNP-loaded nanoparticles

**Fig. 5:**
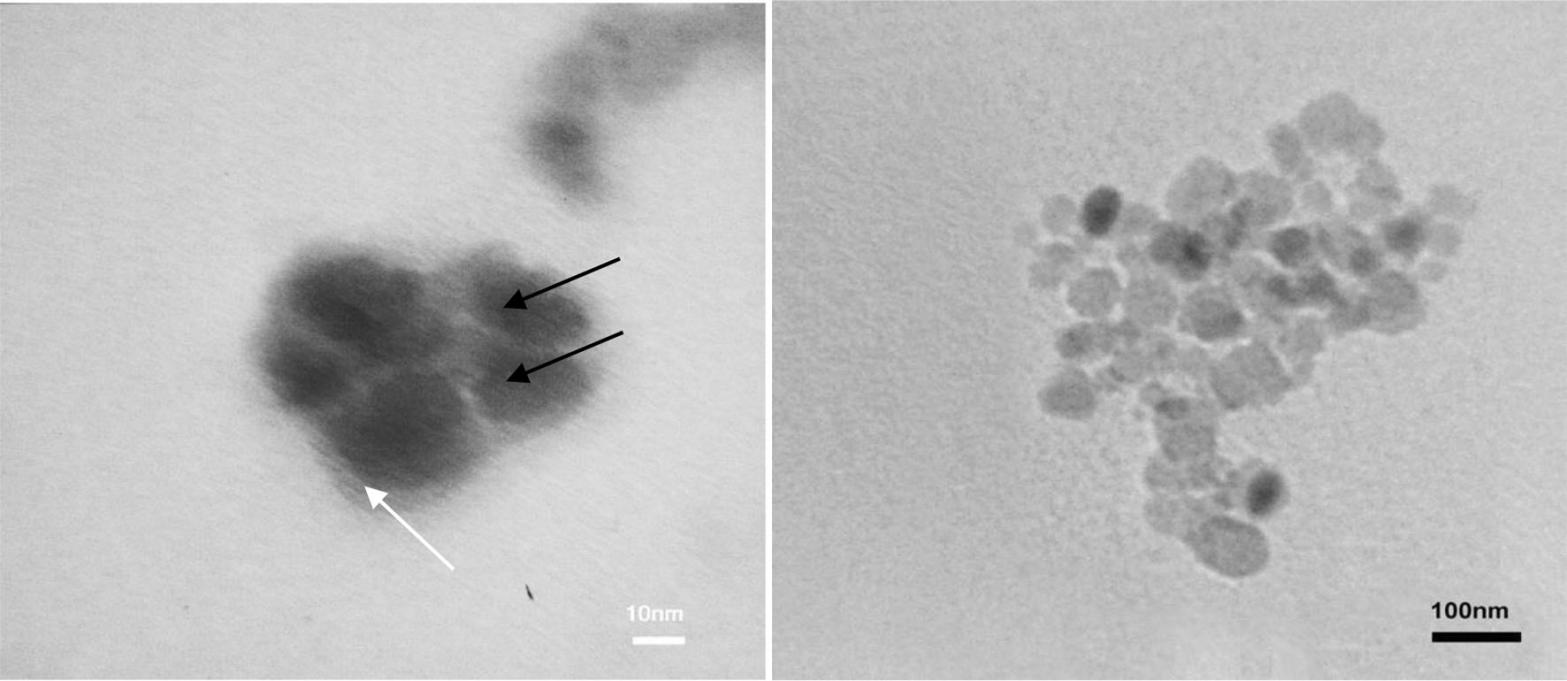
TEM images of the DMNPs: black arrows show the MNPs encapsulated in dextran-spermine polymer, and the white arrow shows the polymer

Superparamagnetic materials have the ability to become magnetized upon exposure to a magnetic field without showing permanent magnetization once removed from the field. This ability is used in nanomedicine as an efficient tool to move nanoparticles into the body towards target sites (Tampieri et al. 2012). TEM imaging and DLS analysis were used to evaluate the average size, size distribution, morphology and encapsulation of the nanoparticles. The zeta potential of the nanoparticles was also measured. As shown in Table 1, the mean size of the nanoparticles was 67.3 nm with zeta potential of +30 mV. In cancer therapeutics, nanoparticles smaller than 200 nm are desirable (Yallapu et al. 2011) and nanoparticles with the hydrodynamic diameter of 10–100 nm are considered optimal for biomedical applications (Cole et al. 2011). The TEM images in Fig. 5 show DMNPs with 60–80 nm size and good encapsulation of MNPs in dextran-spermine network.

**Table 1:** Comparison of average size and zeta potential of the DMNPs, the antibody and the ADMNPs

Sample	Average size (nm)	Zeta potential (mV)
Dextran-spermine encapsulated Iron oxide nanoparticles (DMNPs)	67.3	+30
Anti-HER2 antibody	7.5	−12.5
Anti-HER2 conjugated dextran-spermine encapsulated iron oxide nanoparticles (ADMNPs)	85.6	+3.47

### Characterization of the antibody-conjugated nanoparticles

DLS analysis was carried out to confirm the conjugation of the antibody to DMNPs. As reported in Table 1, the mean size of ADMNPs and the antibody was 85.6 and 7.5 nm, respectively, with corresponding zeta potentials of +3.47 and −12.5 mV. These particles are still within optimal size range and with a cationic surface charge, can be easily uptaken by cancer cells. These results indicate that the average size of DMNPs increased upon antibody conjugation; but the extent of their positive charge decreased due to the negative charge of the antibody. These observations confirm the successful conjugation of the antibody to the DMNPs.

Bradford assay was also used to quantitatively evaluate the antibody conjugation. A standard absorbance curve was plotted for the antibody concentration. The supernatant of the centrifuged antibody-conjugated nanoparticles suspension was analyzed using Elisa reader and conjugation of 24.1 µg antibody to 1 mg of the nanoparticles was obtained. Higher antibody conjugation that resulted in negatively charged nanoparticles have been reported; but these negatively charged nanoparticles did not show good cell uptake characteristics (Nahta and Esteva 2006; Purushotham and Ramanujan 2010; Rao et al. 2013).

### Cytotoxicity assay

**Fig. 6:**
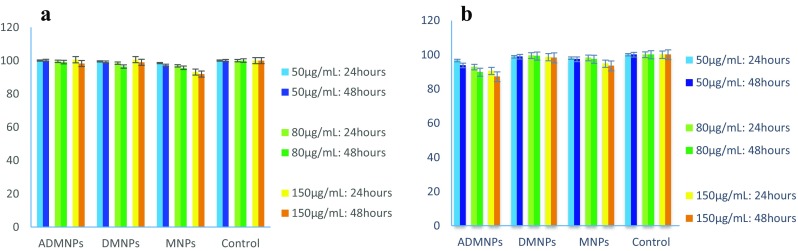
Cytotoxicities of various concentrations and incubation times of ADMNPs, DMNPs and MNPs measured by viabilities of **a** fibroblast cells and **b** SKBR3 calls relative to non-toxic control

Prior to application of any biomaterial in therapy, its potential toxic effects to cells should be evaluated (Dennis et al. 2009). The standard MTT assay was used to determine the cytotoxic effects of MNP, DMNP and ADMNP on SKBR3 and fibroblast cells. The cells were treated by various concentrations (50, 80 and 150 µg/mL) of different nanoparticles and their viabilities after 24 and 48 h were compared to zero-time viability (Fig. 6). For each cell type, a control group without nanoparticle was considered as a reference and the viability values were normalized to the control value. The results confirmed that nanoparticle groups were not cytotoxic to fibroblast cells except for the MNP group which decreased the cell viability slightly after 24 and 48 h of incubation. The MNP cytotoxicity increased at higher concentrations, but still at worst case, with the highest concentration (150 µg/mL) and after 48 h, less than 9% cytotoxicity was observed, so MNPs can be considered nontoxic to fibroblast cells at these concentrations. Previous studies indicated that coated MNPs were not toxic at low concentrations (Easo and Mohanan 2013; Sadhasivam et al. 2015). Encapsulating the MNPs in the dextran-spermine polymer allow them to be used at higher concentrations. The same can be said for SKBR3 cells; the cytotoxic effect of MNPs on the SKBR3 cells was negligible and less than 10%. However, the DMNP group had absolutely no toxic effect on the cancer cells, as the cell viability after 48 h was at the same level as the control group. Since dextran is a hydrophilic, water-soluble polymer that is inert in biological systems (İmren et al. 2006), dextran-encapsulated MNPs were not toxic. Coated MNPs have been reported to be non-toxic to SKBR3 cells (Easo and Mohanan 2013; Sadhasivam et al. 2015). The ADMNPs induced a 13% decrease of the viability after 48 h that is due to anti-proliferative effect of herceptin to HER-2 expressing cells (Chen et al. 2009; Nahta and Esteva 2006).

### Uptake assay

**Fig. 7:**
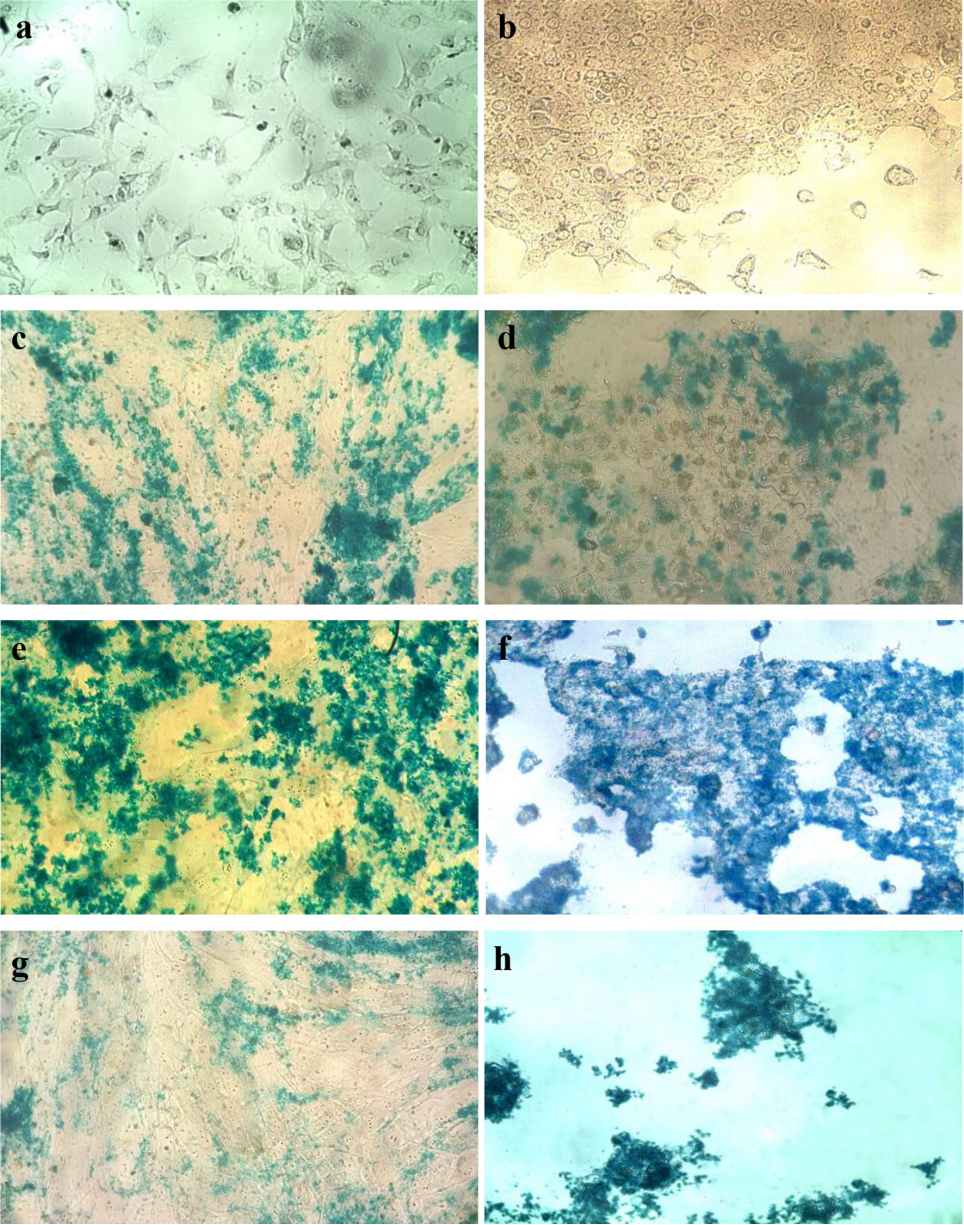
Iron staining results **a** human fibroblast cells (blank), **b** SKBR3 cells (blank), **c** human fibroblast cells stained by MNPs, **d** SKBR3 cells stained by MNPs, **e** human fibroblast cells stained by DMNPs, **f** SKBR3 cells stained by DMNPs, **g** human fibroblast cells stained by ADMNPs and **h** SKBR3 cells stained by ADMNPs

Targeting ability is a must when it comes to cancer treatment, since cancer therapeutics are toxic not only to cancer cells, but also to normal healthy tissues. As for hyperthermia, heating healthy tissues might be harmful; thus, the nanoparticles should be delivered exactly to the tumor site and nowhere else. Iron staining method was used to evaluate the targeting ability of the ADMNP, DMNP and MNPs. The SKBR3 and fibroblast cells were treated by 80 µg/mL magnetic nanoparticles; the cells were washed after 6 h of incubation and the iron particles were stained using Prussian blue. The microscopic images of the cells in Fig. 7 show that ADMNPs have entered the cancer cells with a great number and covered the cells completely, but no blue stain was observed in fibroblast cells. The results for DMNPs showed no difference between cancer and normal cells. These particles having positive surface charge are capable of entering cells, but as they do not have any specific receptor, do it with no preference and would not be a good choice when it comes to hyperthermia treatment. Finally, the MNPs showed less cellular uptake by the cells. These particles with low uptake and no targeting ability cannot be considered as a suitable candidate for hyperthermia. The ADMNPs with good targeting ability and cellular uptake can be considered to be the most promising carrier for cancer hyperthermia.

### Hyperthermia study

**Fig. 8:**
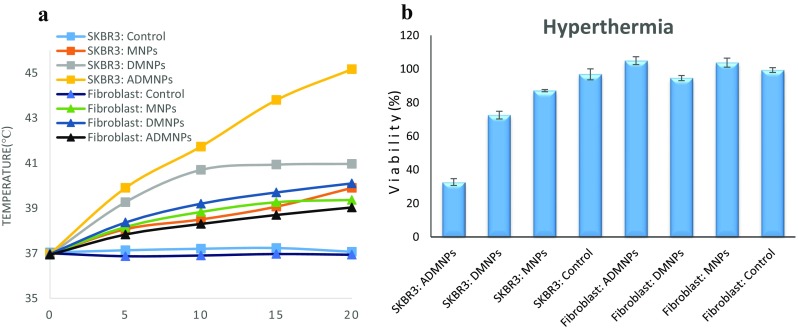
Hyperthermia study of SKBR3 and fibroblast cells: **a** temperature elevations of SKBR3 and fibroblast cells by heating effects of ADMNPs, DMNPs and MNPs under AFM, and **b** viabilities of SKBR3 and fibroblast cells containing ADMNPs, DMNPs and MNPs after a 20-min hyperthermia course relative to untreated control group

In vitro hyperthermia studies were done under AC magnetic field on SKBR3 and fibroblast cells. The cells were seeded in a 96-well plate and each was treated by 80 µg/ml of 3 nanoparticle groups (MNP, DMNP and ADMNP); a control group was also considered. These samples were subjected to the magnetic field for 20 min. In the presence of an alternating magnetic field (AFM), iron oxide magnetic nanoparticles generate heat and can induce hyperthermia (Dennis et al. 2009; Kossatz et al. 2015). The temperature elevation for each group is shown in Fig. 8a. After the hyperthermia test, the cells were incubated for 24 h prior to undergoing MTT assay to evaluate cell viability. The cell viability of the groups after the hyperthermia test is shown in Fig. 8b.

As shown in Fig. 8a, the control groups for SKBR3 and fibroblast cells, having no magnetic content, did not show any change in temperature. The Fibroblast samples did not experience any significant raise of temperature except for the DMNP sample, which came close to the hyperthermia region. The reason is that these cationic nanoparticles with size <100 nm are capable of entering the cells and as they do not have any specific targeting ability (antibody), they enter any cell type indiscriminately (Cole et al. 2011).The MNPs and DMNPs, due to significant uptake into the SKBR3 cells, were able to heat them to 40 and 41 °C, respectively. The most temperature elevation was observed in SKBR3 cells containing ADMNPs. This group is the only one that reached the hyperthermia temperatures, so the most toxicity was expected for this group.

Figure 8b shows that the cytotoxicity in all fibroblast groups was negligible, so none of the groups were able to generate sufficient amount of heat to damage the cells. However, the ADMNPs, having targeting ability, did not enter the fibroblast cells and the cellular uptake of the MNPs and DMNPs was not enough to induce hyperthermia effect. Figure 8a shows that fibroblast groups did not reach the hyperthermia temperatures and no significant cytotoxicity was expected. The only group which was able to kill some of the fibroblast cells was the DMNPs group. Although the uptakes of MNP and DMNP groups were not significantly different for SKBR3 and fibroblast cells, cytotoxicity was higher for cancer cells due to more sensitivity of the cancer cells to high temperatures (Javidi et al. 2015). Finally, the ADMNPs group with excellent targeting ability and good temperature elevation under AFM was able to kill over 65 percent of cancerous cells without affecting normal fibroblast cells.

## Conclusion

Dextran-spermine polymer with 1.43 mM amine groups was successfully synthesized. Magnetic nanoparticles of dextran-spermine (DMNPs) with a size of 67.3 nm and zeta potential of and +30 mV were prepared. These characteristics make the particles ideal for biomedical applications. EDC-NHS method was used to conjugate anti-HER2 antibody to DMNPs that resulted in nanoparticles with a size and surface charge of 85.6 nm and +3.47 mV, respectively. These antibody-conjugated magnetic nanoparticles (ADMNPs) were compared to DMNPs and MNPs in terms of cytotoxicity, targeting ability and hyperthermia efficiency. The results showed that ADMNPs were not toxic to normal cells, while they showed a slight toxicity to SKBR3 cells. However, they can target HER2-expressing cancer cells and enter them effectively. In vitro hyperthermia confirmed the ability of ADMNPs in targeting cancer cells and heating them up to hyperthermia range while more than 63% of cancer cells were destroyed over a 20-min treatment course. Based on the in vitro results, ADMNPs have a great potential for breast cancer treatment by hyperthermia.
